# Improved computation of Lagrangian tissue displacement and strain for cine DENSE MRI using a regularized spatiotemporal least squares method

**DOI:** 10.3389/fcvm.2023.1095159

**Published:** 2023-03-16

**Authors:** Sona Ghadimi, Mohamad Abdi, Frederick H. Epstein

**Affiliations:** Department of Biomedical Engineering, University of Virginia, Charlottesville, VA, United States

**Keywords:** DENSE, cardiac MRI, strain, segmental strain, global strain, heart

## Abstract

**Introduction:**

In displacement encoding with stimulated echoes (DENSE), tissue displacement is encoded in the signal phase such that the phase of each pixel in space and time provides an independent measurement of absolute tissue displacement. Previously for DENSE, estimation of Lagrangian displacement used two steps: first a spatial interpolation and, second, least squares fitting through time to a Fourier or polynomial model. However, there is no strong rationale for such a through-time model,

**Methods:**

To compute the Lagrangian displacement field from DENSE phase data, a minimization problem is introduced to enforce fidelity with the acquired Eulerian displacement data while simultaneously providing model-independent regularization in space and time, enforcing only spatiotemporal smoothness. A regularized spatiotemporal least squares (RSTLS) method is used to solve the minimization problem, and RSTLS was tested using two-dimensional DENSE data from 71 healthy volunteers.

**Results:**

The mean absolute percent error (MAPE) between the Lagrangian displacements and the corresponding Eulerian displacements was significantly lower for the RSTLS method vs. the two-step method for both x- and y-directions (0.73±0.59 vs 0.83 ±0.1, *p* < 0.05) and (0.75±0.66 vs 0.82 ±0.1, *p* < 0.05), respectively. Also, peak early diastolic strain rate (PEDSR) was higher (1.81±0.58 (s-1) vs. 1.56±0. 63 (s^-1^), *p*<0.05) and the strain rate during diastasis was lower (0.14±0.18 (s^-1^) vs 0.35±0.2 (s^-1^), *p* < 0.05) for the RSTLS vs. the two-step method, with the former suggesting that the two-step method was over-regularized.

**Discussion:**

The proposed RSTLS method provides more realistic measurements of Lagrangian displacement and strain from DENSE images without imposing arbitrary motion models.

## Introduction

Many myocardial strain imaging methods are referred to as “tracking” methods, such as speckle tracking, tag tracking, and feature tracking, as they employ image processing techniques to track specific patterns or image features through a time sequence of images by searching for the most probable correspondence from one frame to the next (1). Harmonic phase (HARP) imaging is also a feature-tracking method as it tracks pixels of a common phase from frame to frame (2). In contrast, displacement encoding with stimulated echoes (DENSE) (3) provides fundamentally different data than tracking-based methods, and, accordingly, displacement and strain analysis of DENSE images do not involve tracking features from frame to frame. In cine DENSE (4), the phase of each pixel of the myocardium (or other tissue of interest) in space and time provides an independent measurement of absolute tissue displacement relative to the time when the initial displacement-encoding pulses were applied, typically upon detection of the ECG R-wave. In other words, DENSE intrinsically measures the Eulerian displacement of each pixel. To compute the Lagrangian displacement, where one can observe the pathline of an element of myocardium starting from the beginning of the cardiac cycle as it moves through time, displacement field estimation can be formulated as a regularized least squares minimization problem seeking to find the Lagrangian displacement field with the least mean squared error relative to the measured Eulerian displacements subject to regularization to reduce the effects of noise in the measurements.

**Figure 1:**
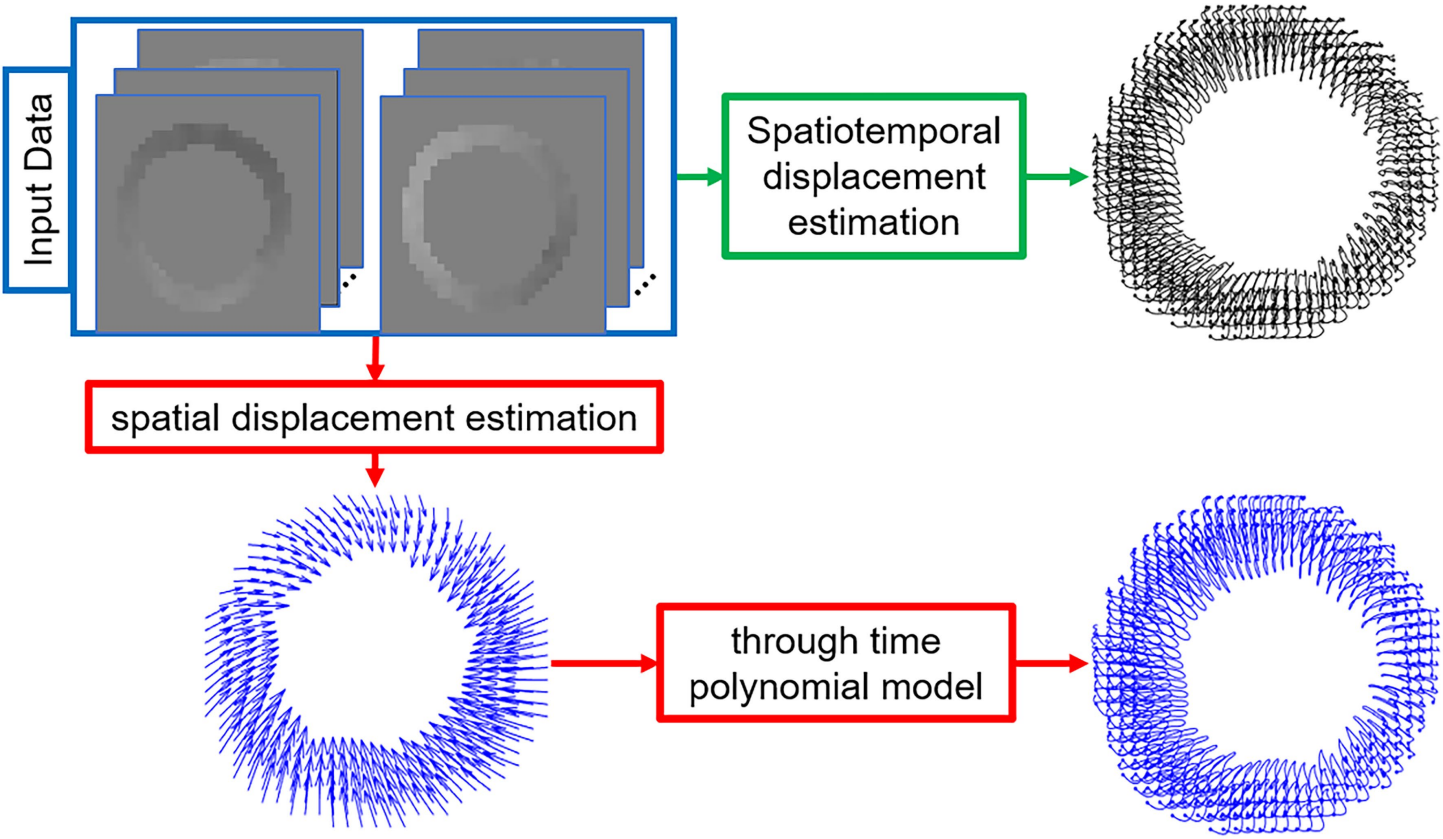
Schematic diagrams of the proposed regularized spatiotemporal least squares method (green path) and the commonly used two-step method (red path) to estimate Lagrangian displacement trajectories from DENSE phase images. The input images are the segmented and phase-unwrapped x- and y-encoded cine DENSE phase images and the output is the left-ventricular myocardial Lagrangian displacement trajectories.

Previously for DENSE, Lagrangian displacement estimation used two steps, including a first step of spatial interpolation or application of a spatial model (spatial interpolation with spatial regularization) combined with a second step of least squares fitting through time to fifth-order Fourier basis functions or a polynomial model (5–10). Alternatively, analysis of DENSE data has used spatial interpolation or modeling without exploiting the time dimension, i.e., treating each frame independently of other frames (11). Limitations of these prior methods are that there are no strong rationales for particular temporal models such as fifth-order Fourier basis functions or polynomial functions and that treating the data independently from frame to frame does not exploit temporal information that is available to denoise the resulting Lagrangian displacement and strain. In the present study, we develop a method to compute the Lagrangian displacement (and, subsequently, strain) for cine DENSE that uses a least squares minimization method to enforce fidelity with the acquired Eulerian displacement data while simultaneously providing model-independent regularization in space and time enforcing only spatiotemporal smoothness (Figure 1). Furthermore, we demonstrate improved quantification of cardiac mechanics using the new method compared to the prior two-step method using data from healthy subjects.

## Methods

### Lagrangian displacement estimation

Prior to computing the myocardial Lagrangian displacement, we assume that the myocardial tissue has been segmented, and if phase wrapping occurred, phase unwrapping would be applied such that for each pixel the phase is directly proportional to Eulerian tissue displacement. These steps may require some manual input by a user or may be completed automatically (12). To compute the Lagrangian displacement field from the Eulerian displacement field, we formulate a minimization problem as:


(1) $$\underset{L_{f}}{\mathrm{argmin}}\left({\left\|\left(AL_{f}-E_{f}\right)\right\|}^{2}+{\left\|\lambda \left(BL_{f}\right)\right\|}^{2}+{\left\|\mu \left(L_{f}-L_{f-1}\right)\right\|}^{2}\right)$$

where $L_{f}$ is the desired Lagrangian displacement trajectory field of frame *f*, $E_{f}$ is the Eulerian displacement computed directly from the unwrapped phase of myocardial pixels in frame *f*, and A is the spatial bilinear interpolation matrix. *B* is the operator taking second derivatives in each spatial direction. ${\left\|\left(AL_{f}-E_{f}\right)\right\|}^{2}$ enforces agreement of the Lagrangian displacement with the measured Eulerian displacement data, ${\left\|\left(BL_{f}\right)\right\|}^{2}$ represents regularization enforcing spatial smoothness of the Lagrangian displacement, ${\left\|L_{f}-L_{f-1}\right\|}^{2}$ represents regularization enforcing temporal smoothness of the Lagrangian displacement, and λ and *μ* are the weights for the regularization terms.

To develop a least squares solution to Eq. 1, we rewrite it as:


(2) $$\underset{L_{f}}{\mathrm{argmin}}\left({\left\|\underset{\hat{A}}{\underset{︸}{\left(\begin{array}{c} A\\ \lambda B\\ \mu \end{array}\right)}}L_{f}-\underset{{\hat{E}}_{f}}{\underset{︸}{\left(\begin{array}{c} E_{f}\\ 0\\ \mu \;L_{f-1} \end{array}\right)}}\right\|}^{2}\right)=\underset{L_{f}}{\mathrm{argmin}}\left({\left\|\hat{A}L_{f}-{\hat{E}}_{f}\right\|}^{2}\right),\begin{array}{c} \begin{array}{l} f\in 1,\ldots ,F \end{array}\\ L_{0}=0 \end{array}\;$$

where F is the total number of image frames, and we assumed there is no Lagrangian displacement before the first frame ($L_{0}=0$).

Assuming that $\hat{A}$is a full-rank matrix, the Lagrangian displacement can be computed using a least squares solution of Eq. 2 given by Eq. 3 (refer to Appendix I for a derivation):


(3) $$L_{f}={\left({\hat{A}}^{T}\hat{A}\right)}^{-1}\left({\hat{A}}^{T}{\hat{E}}_{f}\right)\;$$

The minimization problem of Eq. 1 and its solution given by Eq. 3 are applied separately for each displacement-encoding direction when more than one displacement-encoding direction is employed. In the present study, because our datasets are from two-dimensional (2D) DENSE imaging, we confine our Lagrangian displacement estimations to 2D, although, in theory, the method should be applicable to 3D data. To further denoise the resulting Lagrangian displacement, $L_{f}$, as a final step, we apply a moving mean filter in time with a kernel size of three frames. In addition to denoising, this filter will also add temporal smoothing. More implementation details regarding $\hat{A}$ and $\hat{E}$ are provided in Appendix II.

**Figure 2:**
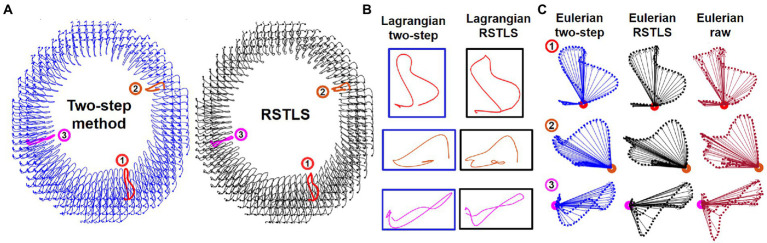
**(A)** An example of 2D Lagrangian displacement trajectories computed using the two-step method with a through-time polynomial function and the proposed regularized spatiotemporal least squares (RSTLS) method from a healthy subject. Panel **(B)** shows three magnified example Lagrangian trajectories computed with both the two-step and RSTLS methods, and panel **(C)** shows the same magnified trajectories projected to the Eulerian domain. The raw Eulerian trajectories (at all discrete time points) calculated directly from the unwrapped DENSE phase data are also shown in **(C)**. These examples demonstrate that abrupt changes in trajectories are over-smoothed using the two-step method but are better depicted using the RSTLS method. The Lagrangian trajectories projected into the Eulerian domain show that RSTLS maintains better agreement than the two-step method with the raw Eulerian measurements.

Figure 2 shows examples of short-axis 2D Lagrangian displacement trajectories computed using the proposed RSTLS method and the prior two-step method that first applies agreement with the measured data and spatial regularization (Eq. 1 with μ=0) and subsequently performs through-time fitting of a 10th order polynomial. Figure 2B shows three magnified example Lagrangian trajectories computed with both the two-step and RSTLS methods, and Figure 2C shows the same magnified trajectories projected to the Eulerian domain. Also shown in Figure 2C are the raw Eulerian trajectories (at all discrete time points) calculated directly from the unwrapped DENSE phase data. Example Lagrangian displacement movies and circumferential strain movies computed using the two-step method and the RSTLS method are shown in the supplementary presentation. These examples illustrate that RSTLS better captures abrupt changes in Lagrangian and Eulerian trajectories and shows better agreement with the raw Eulerian data whereas the two-step method tends to over-smooth the Lagrangian and Eulerian trajectories and shows worse agreement with the raw Eulerian data.

### Data acquisition protocol

For this study, we used two-dimensional (2D) short-axis cine DENSE MRI data acquired from 71 healthy volunteers. All CMR was performed in accordance with a protocol approved by the Institutional Review Board for Human Subjects Research at our institution, and all experiments were performed in accordance with relevant guidelines and regulations. Data were acquired using a 3 T system (Magnetom Prisma, Siemens, Erlangen, Germany). Cine DENSE image acquisition parameters included a pixel size of 3.4 mm^2^ × 3.4 mm^2^, FOV = 200 mm^2^ (using outer volume suppression), slice thickness = 8 mm, a temporal resolution of 15 ms (with view sharing), 2D in-plane displacement encoding using the simple three-point method (13), displacement-encoding frequency = 0.06 or 0.1 cycles/mm, ramped flip angle with a final flip angle of 15°, echo time = 1.08 or 1.26 ms, and a spiral k-space trajectory with 4 interleaves, providing a breath-hold scan time of 14 heartbeats. For most volunteers, cine DENSE images were acquired at basal, mid-ventricular, and apical levels.

### Comparison of displacement, strain, and strain rate computed with the two-step and RSTLS methods

To quantitively evaluate and compare the RSLTS and two-step methods, we first computed the agreement between the computed Lagrangian displacements and the corresponding Eulerian displacements obtained from DENSE unwrapped phase images for each frame. Specifically, the mean absolute percent error (MAPE) was computed as:


(4) $${\left(MAPE\right)}_{f}=\frac{1}{n}\overset{n}{\underset{i=1}{\sum }}\left|\frac{E_{i,f}-AL_{i,f}}{E_{i,f}}\right|$$

where *n* is the total number of myocardial pixels in frame *f*, $L_{i,f}$ is the estimated Lagrangian displacement, and $E_{i,f}$ is the measured Eulerian displacement of pixel *i* in frame *f*. The Lagrangian displacement $L_{i,f}$ is multiplied by the bilinear interpolation matrix A to compute the Eulerian displacement. The purpose of choosing the percent error as opposed to the absolute error was to determine how close an estimated displacement is to a measured displacement regardless of the displacement magnitude, which differs substantially between various cardiac phases.

To investigate the effects of RSTLS vs. the two-step method on strain and strain rate, global (whole-slice) and segmental circumferential strain (E_cc_) and strain rate curves were computed from Lagrangian displacements calculated using both methods. End-systolic strain is widely used as an important metric of systolic function, and strain rate is widely considered an important metric of diastolic function (14–16). The 2D Lagrangian finite strain tensor *E* was computed using Eq. 5


(5) $$E=\frac{1}{2}\left(F^{T}F-I\right)$$

where *I* is the identity matrix and *F* is the deformation gradient tensor. *F* represents the relationship between the myocardium in the undeformed configuration (first frame) and in a deformed configuration (e.g., a phase in cardiac systole). Let the positions of the myocardial points in the undeformed and deformed configurations be *X* and *x*, respectively. Then,


(6) $$dx=F\;dX,F=\left[\begin{array}{cc} \frac{\partial x_{1}}{\partial X_{1}} & \frac{\partial x_{1}}{\partial X_{2}}\\ \frac{\partial x_{2}}{\partial X_{1}} & \frac{\partial x_{2}}{\partial X_{2}} \end{array}\right],\mathrm{and}\;F_{ij}=\frac{\partial x_{i}}{\partial X_{j}}\;i,j\in \left\{1,2\right\}$$

In Eq. 6, *dX* is the position difference between myocardial points in the undeformed (first) frame, which is transformed to *dx* in the deformed frame. Each entry of this tensor ($\;F_{ij}$) is defined as $\partial x_{i}/\partial X_{j}$and determines how the distance between any two elements along the *j*^th^ direction in the reference configuration is projected to the *i*^th^ direction in the deformed configuration. After computing *F* and *E* in the Cartesian coordinate system, strain tensors are mapped to the heart’s short-axis polar coordinate system using the rotation matrix shown in Eq. 7, in which $E_{polar}\left(1,1\right)$ is the radial (*E*_rr_) and $E_{polar}\left(2,2\right)$ is the circumferential strain (*E*_cc_).


(7) $$E_{polar}=R_{rot}ER_{rot}^{T},R_{rot}=\left[\begin{array}{cc} \cos\left(\theta \right) & -\sin\left(\theta \right)\\ \sin\left(\theta \right) & \cos\left(\theta \right) \end{array}\right]$$

Once circumferential strain is calculated for all myocardial points and all time points, the strain rate is obtained by taking the difference of strain values in two consecutive frames divided by the repetition time (TR).

Finally, to explore how the proposed RSTLS method and the two-step method propagate errors in the calculation of Lagrangian displacement, we arbitrarily added simulated measurement errors to a specific pixel in various time frames and visually compared the resulting computed Lagrangian trajectories of that pixel to uncorrupted trajectories. The simulated error displacement vector was perpendicular to the original vector with a magnitude of 50% of the uncorrupted displacement vector.

## Results

**Figure 3:**
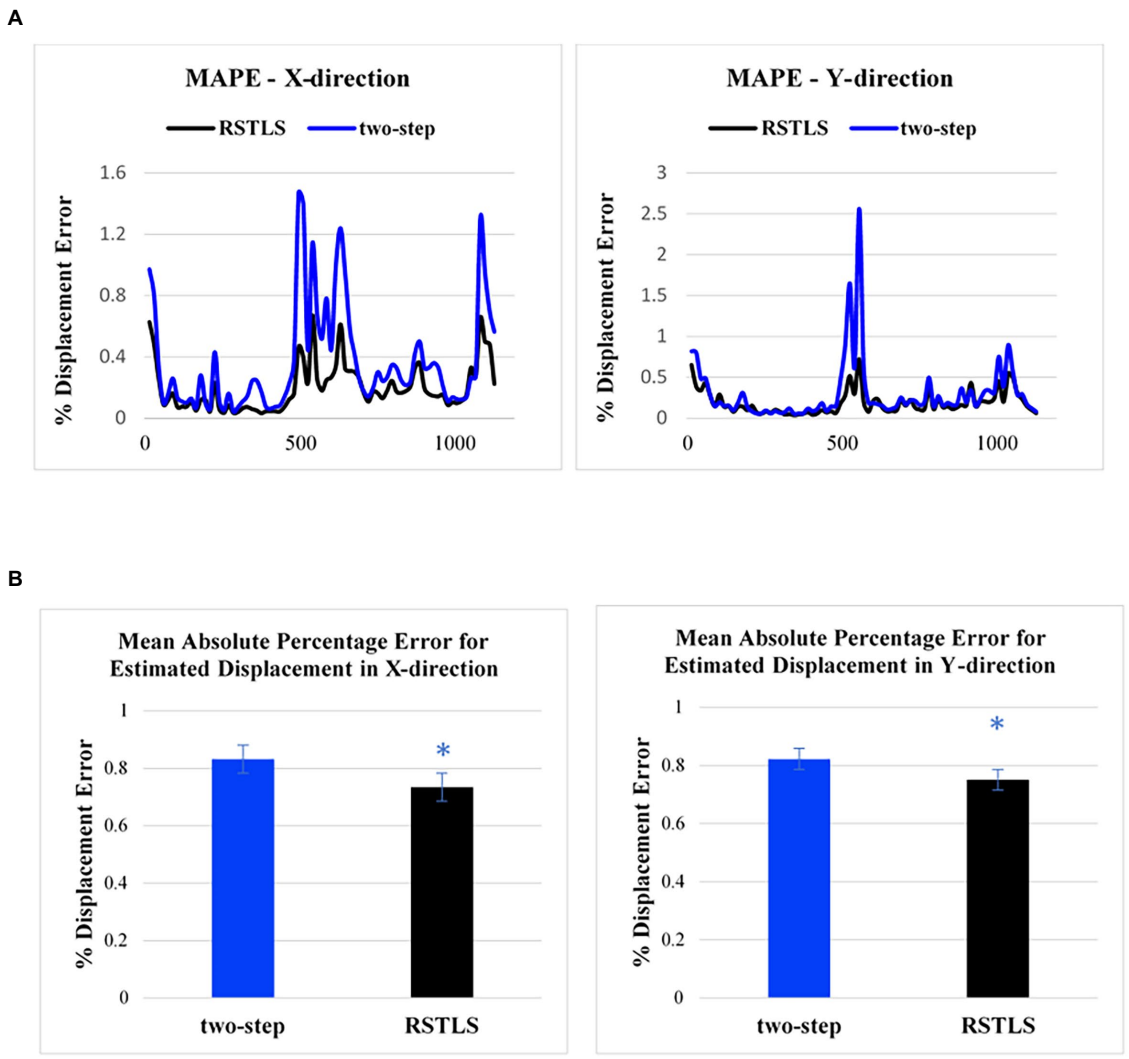
**(A)** Examples of the mean absolute percentage error for the two-step and RSTLS methods as a function of time in the cardiac cycle for displacement measured in the x- and y-directions. The greatest percent of errors appear in early systole and early and late diastole. **(B)** Comparison of mean absolute percentage error for healthy subjects averaged over time. The graphs are plotted based on 182 slices from 71 volunteers. Error bars represent standard error. *p < 0.05.

Figure 3A shows an example of the calculation of MAPE for the RSTLS and two-step methods. While the time of 330 ms represented end systole in this example, the highest MAPE values occurred in early- and late-diastolic phases, some of which have small absolute displacements. Using paired t-tests to compare the MAPE (averaged over time) for Lagrangian displacement computed using the RSTLS and two-step methods for displacement encoding applied in the x- and y-directions, Figure 3B shows that MAPE was significantly lower for the RSTLS method in both the x- and y-directions (0.73 ± 0.59 vs. 0.83 ± 0.1, *p* < 0.05), (0.75 ± 0.66 vs. 0.82 ± 0.1, *p* < 0.05), respectively.

**Figure 4:**
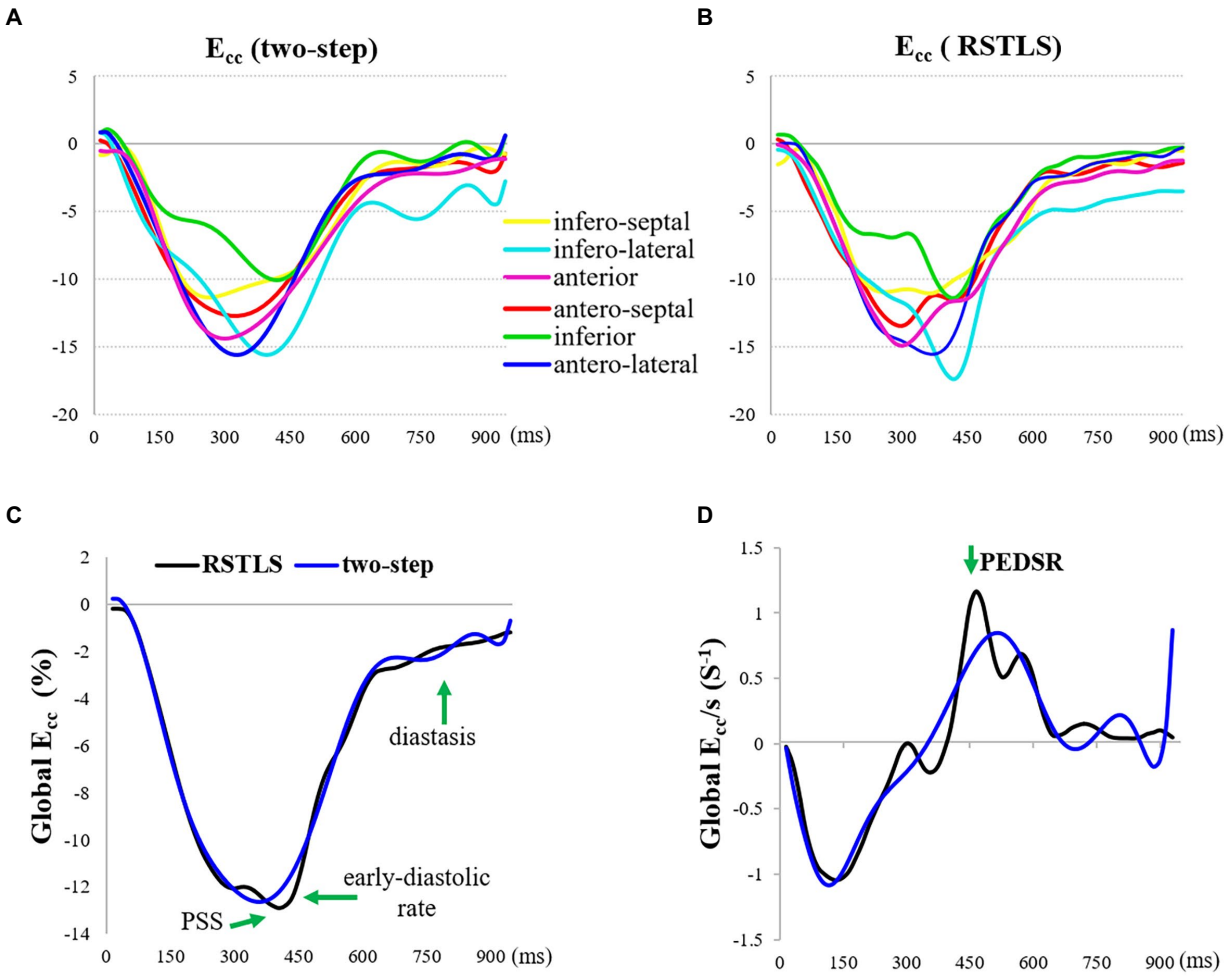
An example from a heathy subject comparing circumferential strain (E_cc_) curves and strain rate (E_cc_/s) curves computed using the two-step and RSTLS methods. In both segmental **(A, B)** and global **(C, D)** assessments, the RSTLS method better captures features such as post-systolic shortening, early diastole, and diastasis, that are over-smoothed or have oscillation artifacts when computed using the two-step method. PSS = post-systolic shortening, and PEDSR = peak early diastolic strain rate.

**Figure 5:**
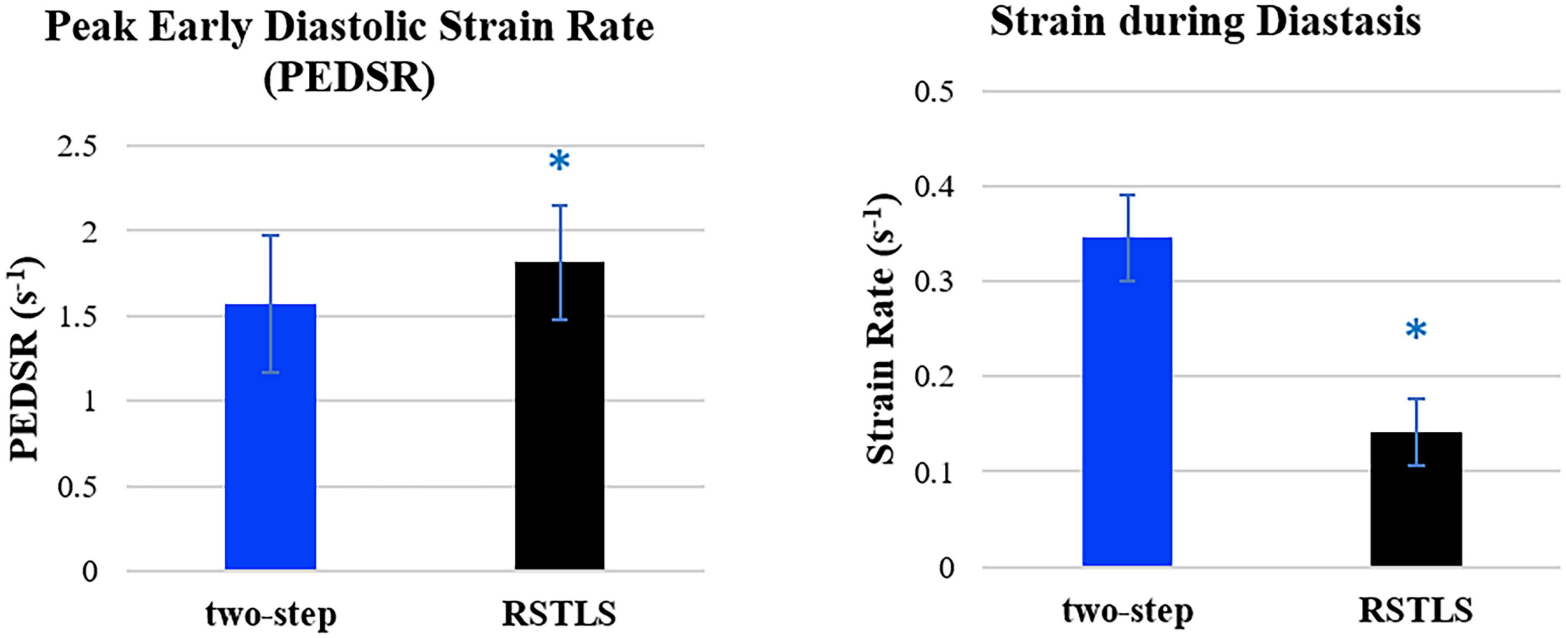
Comparison of global peak early diastolic strain rate and strain rate during diastasis for healthy subjects. The graphs are plotted based on 182 slices from 71 volunteers. Error bars represent standard deviations. *p < 0.05.

Figure 4 demonstrates segmental and global E_cc_ strain and strain rate vs. time curves of a healthy volunteer. Qualitatively, it can be observed that RSTLS preserves the visualization of post-systolic shortening, which is over-smoothed by the two-step method. In addition, model-driven strain oscillations that are commonly observed in diastasis using the two-step method, related to the though-time polynomial fit, are avoided using the RSTLS method. To quantify the advantages of RSTLS vs. the two-step method for the analysis of diastole, we computed the peak early diastolic strain rate (PEDSR) and the strain rate during diastasis. For global strain and strain-rate data, Figure 5 shows the mean ± standard deviation of PEDSR and of the strain rate during diastasis. The PEDSR was higher (1.81±0.58 (s^-1^) vs. 1.56±0. 63 (s^-1^), *p* < 0.05) and the strain rate during diastasis was lower (0.14±0.18 (s^-1^) vs 0.35±0.2 (s^-1^), *p* < 0.05) for the RSTLS vs. the two-step method. The higher PEDSR likely reflects less temporal over-smoothing, and the lower diastasis strain rate likely reflects the absence of model-induced oscillation artifacts for the RSTLS method.

**Figure 6:**
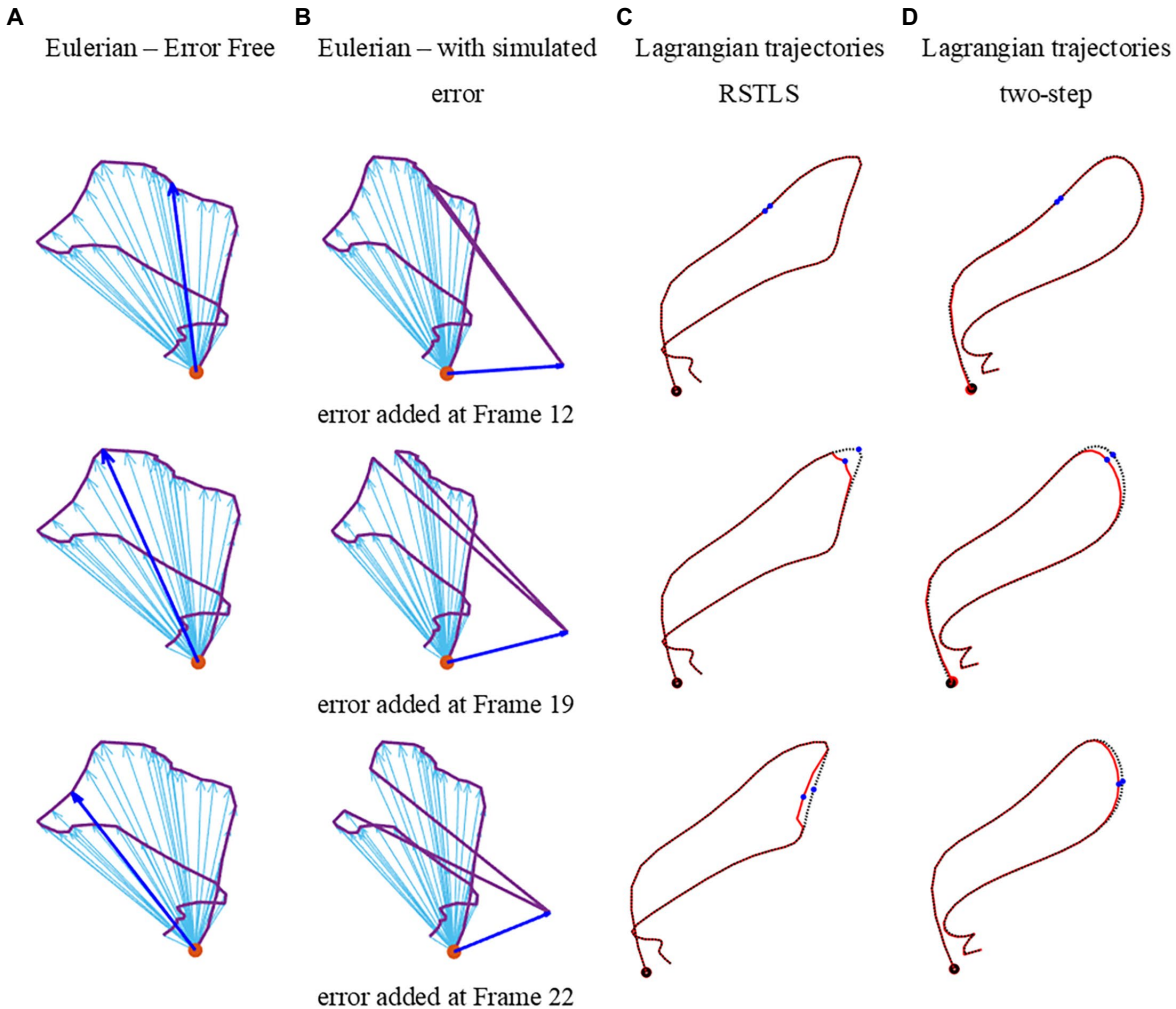
Examples of errors added to measured Eulerian displacements and their effects on computed Lagrangian displacements. **(A)** depicts the uncorrupted Eulerian displacements derived from DENSE unwrapped phase data from one pixel at all time points. The dark blue arrow corresponds to the Eulerian displacement of the time frame selected for adding a simulated measurement error. The new corrupted Eulerian displacements with simulated measurement errors are shown in **(D)**, where the error displacement vector (blue vector) is perpendicular to the original vector. Panel **(C)** shows the computed Lagrangian trajectories using the RSTLS method without and with the simulated error (the dotted black line shows the uncorrupted Lagrangian trajectory and the solid red line shows the corrupted Lagrangian trajectory). The blue dots in the Lagrangian trajectories show the time points where the simulated error was inserted. Panel **(D)** is the same as **(C)**, except it was generated using the two-step method instead of the RSLTS method for computing the Lagrangian trajectories.

Figure 6 illustrates how errors added to the specific pixel at different time frames propagate through the Lagrangian displacement calculations. Figure 6A depicts the uncorrupted Eulerian displacement derived from DENSE unwrapped phase data from one pixel at all time points. The dark blue arrow corresponds to the Eulerian displacement of the time frame selected for adding a simulated measurement error. The new corrupted Eulerian displacement with a simulated measurement error is shown in Figure 6B, where the error displacement vector (blue vector) is perpendicular to the original vector. Next, Figure 6C shows the computed Lagrangian trajectories using the RSTLS method without and with the simulated error (the dotted black line shows the uncorrupted Lagrangian trajectory and the solid red line shows the corrupted Lagrangian trajectory). The blue dots in the Lagrangian trajectories show the time point where the simulated error was inserted. Figure 6D is the same as Figure 6C, except it was generated using the two-step method instead of the RSLTS method for computing the Lagrangian trajectories. For both the two-step and RSTLS methods, these computations show that when errors occur, instead of leading to further and increasing errors through time along the trajectory, both methods demonstrate a self-correction property, as the trajectories quickly return to their uncorrupted form within just a few time frames after the errors were inserted.

## Discussion

**Figure 7:**
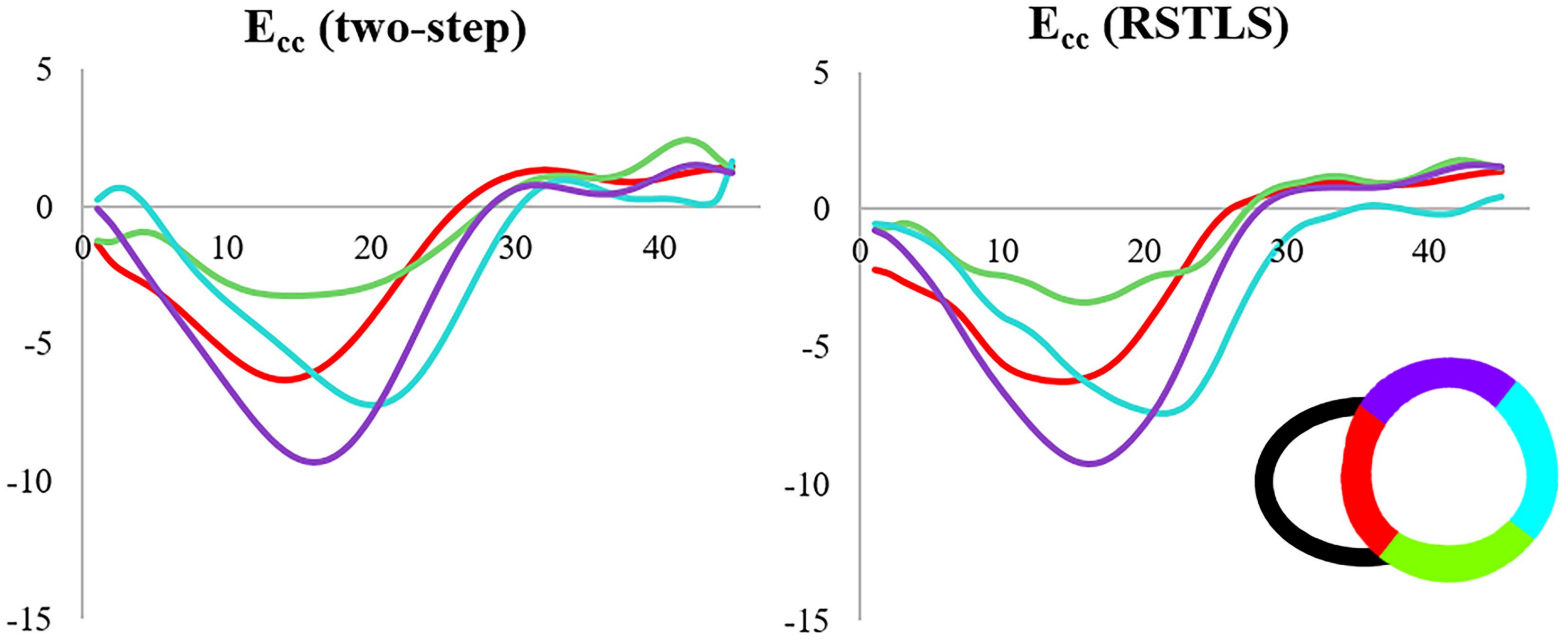
Example demonstrating artifactual pre-stretch in early systole in the apical-lateral wall using the two-step method that is not observed using the RSTLS method from a patient with ischemic heart disease.

We have developed an improved approach to estimate Lagrangian displacement and strain from DENSE phase images that supports spatial and temporal regularization, enforces fidelity with the acquired Eulerian displacement data, and does not impose model-based assumptions on the displacement solution. While our experiments did not include a gold standard reference for measurements of cardiac mechanics, comparisons with the prior two-step displacement estimation method suggest that RSTLS provides a better depiction of the strain–time curve, particularly with regard to post-systolic shortening, early diastolic strain, and diastasis. Specifically, the proposed RSTLS method likely avoids temporal over-smoothing and model-driven oscillations. The model-driven oscillations are often seen in diastasis (as in Figure 4), but they can also manifest in other ways, such as false prestretch at the beginning of the cardiac cycle, as shown in Figure 7.

An important characteristic of the RSTLS method is that it processes spatiotemporal DENSE data in an integrated fashion, as opposed to separately processing the spatial and temporal data. Prior methods for the analysis of DENSE phase images first perform spatial interpolation, and subsequently model the through-time dimension or simply apply the spatial calculations to each frame independent of other frames. These other methods include the radial point interpolation method described by Kar et al. (17), another finite element method described by Young et al. (8), and our previous study that first performed spatial interpolation and then handled the time dimension (6). To the best of our knowledge, the present RSTLS method is the first to treat the 2D + time DENSE data in a unified way.

Our investigations showed that measurement errors introduced at one cardiac phase do not propagate to subsequent cardiac phases, but instead the calculation of the Lagrangian trajectory has a self-correction property. This property occurs because fidelity with the acquired data is enforced for each cardiac phase. This is an important property of DENSE and is an advantage compared to traditional block-matching methods, where an error in one phase can propagate and accumulate over time (18, 19).

Applications, where RSTLS may have advantages, include the assessment of late mechanical activation in patients who may be candidates for cardiac resynchronization therapy (20), as model-driven oscillations in the strain–time curve using the two-step method can mimic early stretch in the circumferential direction (as shown in Figure 6), and RSTLS avoids these model-driven oscillations. In addition, due to the advantages of RSTLS for measuring diastolic strain rate, this method may be preferred when the assessment of diastolic function is important such as for heart failure with preserved ejection fraction (14) and pulmonary hypertension (15).

Our study had limitations. First, we confined our investigations to two dimensions, while, in reality, myocardial displacement and strain are three-dimensional. In the future, the RSTLS method can be extended to three dimensions. Second, we did not include patient data in our analysis and also there is a lack of multi-center validation in this study. Third, while in Appendix II, we give recommendations for suggested values of the regularization weights, μ and λ, based on our experience applying RSTLS in human subjects (21, 22), we have not provided a rigorous optimization of these values. To rigorously optimize μ and λ, ideally, we would make use of computer simulations with known displacements that closely match *in vivo* myocardial mechanics, both spatially and temporally. We could then apply RSTLS to simulated DENSE data and identify values of μ and λ that lead to RSTLS estimates of Lagrangian displacement that most closely agree with the known values. However, while very good models exist for mimicking the spatial mechanics of the heart (23), to the best of our knowledge, there are no such models that comprehensively mimic spatiotemporal myocardial mechanics. Since RSTLS incorporates spatiotemporal motion, the lack of an appropriate spatiotemporal model is an obstacle to rigorous optimization of the regularization weights. Finally, Eq. 1, which describes the RSTLS model, utilizes the one-sided first-order difference for temporal regularization, whereas the central difference would be more accurate for estimating the derivative. We chose the one-sided first-order difference because it facilitates the use of a simple least squares solution to Eq. 1 by Eq. 3. If we instead used the central difference in Eq. 1, the least squares method would not be suitable for solving it and a more complicated iterative method would need to be used to solve the minimization equation.

A disadvantage of the RSTLS method compared to the two-step method may be less robustness to noise. To address this problem, noise reduction filtering with a mean filter was applied after using the RSTLS method to compute the Lagrangian displacement trajectories. Although fitting a through-time polynomial model imposes some artifacts and some over-smoothing, the two-step method may provide better results for very noisy data.

## Conclusion

The present study developed an approach to estimate Lagrangian displacement from DENSE images using a regularized spatiotemporal least squares method. Evaluations using images from healthy subjects demonstrated that the RSTLS method combines spatial regularization, temporal regularization, and agreement with measured Eulerian displacement to provide a model-free computation of myocardial Lagrangian displacement and strain that provides reduced mean absolute percent error and higher peak early diastolic strain rate, suggesting better accuracy and less over-regularization compared to the competing two-step method.

## Data availability statement

Requests to access these datasets should be directed to fhe6b@virginia.edu.

## Ethics statement

The studies involving human participants were reviewed and approved by University of Virginia Institutional Review Board. The patients/participants provided their written informed consent to participate in this study.

## Author contributions

SG developed the methods, analyzed data, and helped draft the manuscript. MA acquired some of the healthy volunteer data. FE provided the intellectual background necessary and helped draft the manuscript. All authors contributed to the article and approved the submitted version.

## Funding

This work was supported by the National Institute of Health (NIH), and the National Heart, Lung, and Blood Institute (NHLBI) Research Project (R01) R01 HL147104.

## Conflict of interest

The authors declare that the research was conducted in the absence of any commercial or financial relationships that could be construed as a potential conflict of interest.

## Publisher’s note

All claims expressed in this article are solely those of the authors and do not necessarily represent those of their affiliated organizations, or those of the publisher, the editors and the reviewers. Any product that may be evaluated in this article, or claim that may be made by its manufacturer, is not guaranteed or endorsed by the publisher.

## Abbreviations

DENSE: Displacement Encoding with Stimulated Echoes
MRI: Magnetic resonance imaging
ECG: Electrocardiogram
LV: Left ventricular
RSTLS: Regularized spatiotemporal least squares
MAPE: Mean absolute percent error
PEDSR: Peak early diastole strain rate
Ecc: Circumferential strain
