# Supplementary material for: Improved computation of Lagrangian tissue displacement and strain for cine DENSE MRI using a regularized spatiotemporal least squares method
Source: Front Cardiovasc Med. 2023 Mar 16;10:1095159. doi: 10.3389/fcvm.2023.1095159 (PMC10061004; doi:10.3389/fcvm.2023.1095159)
Supplement: Supplementary file 1 [file Data_Sheet_1.docx]

**Appendix I:**

As shown in the Methods section, to compute the Lagrangian displacement field from the Eulerian displacement field, we formulate a minimization problem as:

$$\underset{L_{f}}{\mathrm{argmin}} \left( \left\| \left( AL_{f}-E_{f} \right) \right\|^{2}+\left\| \lambda\left( BL_{f} \right) \right\|^{2}+\left\| \mu\left( L_{f}-L_{f-1} \right) \right\|^{2} \right)$$

To develop a least squares solution, we rewrite it as:

$$=\underset{L_{f}}{\mathrm{argmin}} \left( \left\| \underset{\hat{A}}{\underbrace{\left( \begin{aligned} A \\ \lambda B \\ \mu\end{aligned} \right)}}L_{f}-\underset{\hat{E}_{f}}{\underbrace{\left( \begin{aligned} E_{f} \\ 0 \\ \mu L_{f-1} \end{aligned} \right)}} \right\|^{2} \right)$$

which can be further rewritten as

$$=\underset{L_{f}}{\mathrm{argmin}} \left( \left\| \hat{A}L_{f}-\hat{E}_{f} \right\|^{2} \right)$$

where $E_{f}$ is the Eulerian displacement computed directly from the unwrapped phase of myocardial pixels in frame *f*, and $L_{f-1}$ is the computed Lagrangian displacement trajectory field from frame *f-1*. $f\in1,\ldots, F$ and we assume $L_{0}=0.$

It should be noted that the Lagrangian displacement is estimated by solving the minimization problem for each frame independently. The only term related to the previous frame is the estimated Lagrangian displacement vector, $L_{f-1}$. For the first frame we have assumed $L_{0}=0$. Details for computing $\hat{A}$ and $\hat{E}_{f}$ are provided in Appendix II.

We solve for $L_{f}$ by minimizing the energy of the error:

$$J\left( L_{f} \right)=\left\| \hat{E}_{f}-\hat{A}L_{f} \right\|_{2}^{2}$$

Before solving this problem by least squares, all trajectory field maps (Eulerian and Lagrangian) have to be vectorized. Expanding $J\left( L_{f} \right)$ gives,

$$J\left( L_{f} \right)=\left( \hat{E}_{f}-\hat{A}L_{f} \right)^{T}\left( \hat{E}_{f}-\hat{A}L_{f} \right)$$

$$={\hat{E}_{f}}^{T}\hat{E}_{f}-{\hat{E}_{f}}^{T}\hat{A}L_{f}-{L_{f}}^{T}\hat{A}^{T}\hat{E}_{f}+{L_{f}}^{T}\hat{A}^{T}\hat{A}L_{f}$$

Note that each of the four terms in the above equation are scalars and the transpose of a scalar is the same scalar, such that ${\hat{E}_{f}}^{T}\hat{A}L_{f}= {{{(\hat{E}}_{f}}^{T}\hat{A}L_{f})}^{T}$

Using this, we can rewrite $J\left( L_{f} \right)$ as:

$$J\left( L_{f} \right)= {\hat{E}_{f}}^{T}\hat{E}_{f}-2{\hat{E}_{f}}^{T}\hat{A}L_{f}+{L_{f}}^{T}\hat{A}^{T}\hat{A}L_{f}$$

Taking the derivative, gives

$$\frac{\partial}{\partial L_{f}}J\left( L_{f} \right)=-2\hat{A}^{T}\hat{E}_{f}+2\hat{A}^{T}\hat{A}L_{f}$$

Setting the derivative to zero, gives

$\frac{\partial}{\partial L_{f}}J\left( L_{f} \right)=0 \underset{\Rightarrow}{}$ $\hat{A}^{T}\hat{A}L_{f}=\hat{A}^{T}\hat{E}_{f}$

Finally, assuming $\hat{A}^{T}\hat{A}$ is invertible, the solution is given by

$$L_{f}={(\hat{A}^{T}\hat{A})}^{-1}\left( \hat{A}^{T}\hat{E}_{f} \right), \hat{A}= \left( \begin{aligned} A \\ \lambda B \\ \mu\end{aligned} \right), \hat{y}_{f}= \left( \begin{aligned} E_{f} \\ 0 \\ \mu L_{f-1} \end{aligned} \right)$$
