# Supplementary material for: Improved computation of Lagrangian tissue displacement and strain for cine DENSE MRI using a regularized spatiotemporal least squares method
Source: Front Cardiovasc Med. 2023 Mar 16;10:1095159. doi: 10.3389/fcvm.2023.1095159 (PMC10061004; doi:10.3389/fcvm.2023.1095159)
Supplement: Supplementary file 2 [file Data_Sheet_2.docx]

**Appendix II:**

The Eulerian vectors in a DENSE displacement field at any time frame ($t_{f}=t_{1},\ldots,t_{F}$) reference the corresponding tissue position at time $t_{0}$. With this information, the Eulerian displacement vector origin ($P_{0}$) can be computed for each frame as $P_{0}=\left[ \begin{matrix} i-E_{x_{f}} \\ j-E_{y_{f}} \end{matrix} \right]$, where *i* and *j* are image grid points, and $E_{x_{f}}$ and $E_{y_{f}}$ are the corresponding Eulerian displacements for frame, *f*, in the *x*- and *y*-directions, respectively. In case of 3D DENSE, a third row will be added corresponding to the *z*-direction.

Using the RSTLS formulation of Eq. 4, as discussed in appendix I, the Lagrangian displacement $L_{f}=\left[ \begin{matrix} L_{x_{f}} \\ L_{y_{f}} \end{matrix} \right]$ is computed by:

$$L_{f}={(\hat{A}^{T}\hat{A})}^{-1}\left( \hat{A}^{T}\hat{E}_{f} \right), \hat{A}= \left[ \begin{aligned} A \\ \lambda B \\ \mu\end{aligned} \right], \hat{y}_{f}= \left[ \begin{aligned} E_{f} \\ 0 \\ \mu L_{f-1} \end{aligned} \right]$$

To implement above equation, we modified methods described by D’Errico [23].

$\hat{\boldsymbol{A}}$ **computation:**

To compute the Lagrangian displacement of a point on the grid at time t_0_, the first step is to define the interpolation method. For 2D DENSE we used bilinear interpolation and for 3D DENSE we used trilinear interpolation.

| 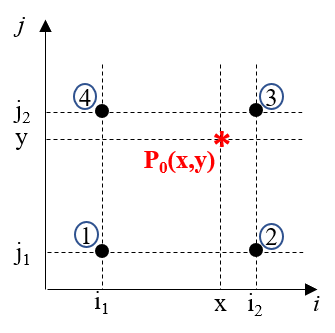 |
| --- |
| Figure A.1. Bilinear interpolation using 4 neighbors. |

As DENSE provides the origin of each Eulerian displacement measurement, *P*_0_(*x*,*y*) (Figure A.1), the interpolation equation is computed as:

$$E_{P0}\left( x,y \right)=\left( 1-t_{x} \right)\left( 1-t_{y} \right)L_{1}+t_{x}\left( 1-t_{y} \right)L_{2}+t_{x}t_{y}L_{3}+\left( 1-t_{x} \right)t_{y}L_{4}$$

where *E_P0_* is the Eulerian displacement for the origin point *P*_0_(*x*,*y*), and *L_1_*,…,*L_4_* are the unknown Lagrangian displacements of the four nearest neighbor grid points defined as

$L_{1}=L(i_{1},j_{1})$, $L_{2}=L(i_{2},j_{1})$, $L_{3}=L(i_{2},j_{2})$, $L_{4}=L(i_{1},j_{2})$.

Also, $t_{x}=\frac{x-i_{1}}{i_{2}-i_{1}} and t_{y}=\frac{y-j_{1}}{j_{2}-j_{1}}$ where *P*_0_ is enclosed by $i_{1}$ and $i_{2}$ in the *x*-direction ($i_{1}<x<i_{2}$) and $j_{1}$ and $j_{2}$ in the *y*-direction ($j_{1}<y<j_{2}$).

$E_{P0}\left( x,y \right)$ can be written in matrix form as:

$$E_{P0}\left( x,y \right)=A_{1\times ngrid}\mathbf{L}_{ngrid\times1},$$

$$where the elements a_{i,j}\mathrm{of} A= \left\{ \begin{aligned} \left( 1-t_{x} \right)\left( 1-t_{y} \right) i=i_{1}, j=j_{1} \\ t_{x} \left( 1-t_{y} \right) i=i_{2}, j=j_{1} \\ t_{x}t_{y} i=i_{2}, j=j_{2} \\ \left( 1-t_{x} \right)t_{y} i=i_{1}, j=j_{2} \\ 0 otherwise \end{aligned} \right., and L=\left[ \begin{aligned} L_{1,1} \\ \vdots\\ L_{i,1} \\ L_{1,2} \\ \vdots\\ L_{i,2} \\ \vdots\\ L_{1,j} \\ \vdots\\ L_{i,j} \end{aligned} \right]$$

Then, we can create the interpolation matrix $A_{n\times ngrid}$ for all myocardial tissue points, where $n$ is the total number of myocardial tissue points and $ngrid$ describes the number of points in a rectangle encompassing the myocardium. The rectangle has dimensions of $n_{i}\times n_{j}$, therefore,$ngrid= n_{i}\times n_{j}$. Each row in the A matrix corresponds to one origin point and each column represents the grid nodes. A is a sparse matrix with 4 non-zero values (4 neighbors) in each row.

The next step is to build the **B** matrix which is a spatial regularizer using the second derivative for all grid points. Here, B is not a Laplacian regularizer ($\Delta L=\frac{\partial^{2}L}{{\partial i}^{2}}+\frac{\partial^{2}L}{{\partial j}^{2}}$), instead it is the uncoupled second derivative ($\left[ \begin{aligned} \frac{\partial^{2}L}{{\partial i}^{2}} \\ \frac{\partial^{2}L}{{\partial j}^{2}} \end{aligned} \right]$) that can be calculated using three grid points for each direction. Figure A.2 shows the numerical second derivative equation for grid point (2) using three unevenly spaced points in the *i*-direction.

| 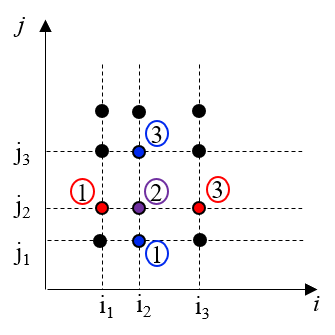 |
| --- |
| Figure A.2. A simple configuration demonstrating the use of unevenly spaced points used to calculate the second derivative of point (2) in the *i*- and *j-*directions for matrix **B**. Red and blue colors show the grid neighbors used in the *i-* and *j-*directions, respectively. |

Referring to Figure A.2,

$$\frac{\partial^{2}L}{{\partial i}^{2}}=\frac{2[{di}_{2}L_{1}-\left( {di}_{1}+{di}_{2} \right)L_{2}+{di}_{1}L_{3}]}{{di}_{1}{di}_{2}({di}_{1}+{di}_{2})}$$

$$\frac{\partial^{2}L}{{\partial j}^{2}}=\frac{2[{dj}_{2}L_{1}-\left( {dj}_{1}+{dj}_{2} \right)L_{2}+{dj}_{1}L_{3}]}{{dj}_{1}{dj}_{2}({dj}_{1}+{dj}_{2})}$$

where ${di}_{2}=i_{3}-i_{2}, {di}_{1}=i_{2}-i_{1}, {dj}_{2}=j_{3}-j_{2}, {dj}_{1}=j_{2}-j_{1}$, and $L_{1}, L_{2}, L_{3}$ are the unknown Lagrangian displacements of 3 nodes.

The sparse B matrix is created for all $ngrid=ni\times nj$ grid points in both the *i* and *j* directions and then concatenated as $B=\left[ \begin{aligned} \frac{\partial^{2}L}{{\partial i}^{2}} \\ \frac{\partial^{2}L}{{\partial j}^{2}} \end{aligned} \right]_{(2\times ngrid)\times ngrid}$.

Finally, for $\hat{A}$, the matrices $A_{n\times ngrid}$, $B_{(2\times ngrid)\times ngrid}$ and $I_{ngrid\times ngrid}$ are concatenated and weighted such that $\hat{A}= \left[ \begin{aligned} A \\ \lambda\alpha B \\ \mu I \end{aligned} \right]$. The values of the spatial and temporal weighting factors λ and μ, respectively, are determined empirically. The λ value controls the spatial smoothness of the estimated surface, and μ controls the temporal smoothness. The last scaling factor is $\alpha$, which is given by $\alpha=\sqrt{\frac{number of rows in matrix A}{number of non-zero rows in matrix B}}$. As this parameter is determined by the A and B matrices, to simplify the equations (1-3) we refer to $\alpha B$ as the $B$ matrix. More information about $\alpha$ can be found in [23,24].

$\hat{E}_{f}$ **computation:**

$\hat{E}_{f}= \left[ \begin{aligned} E_{f} \\ 0 \\ \mu L_{f-1} \end{aligned} \right]$, where ${E_{f}}_{n\times1}$ is the vectorized Eulerian displacement for myocardial tissue points (*n*) in frame $f, f=1,\ldots,F$. $L_{f-1}$ is the calculated Lagrangian displacement of frame *f-1* computed using the RSTLS method. For the initialization, we assume there is no displacement at time $t_{0}$, such that $L_{0}=0.$

**λ and μ weighting factors**

For RSTLS solutions for DENSE myocardial Lagrangian displacements, λ must have a positive value. We empirically determined that, for cine DENSE imaging of the human heart, λ should be in the range [3, 10]. We have found that circumferential and longitudinal strain values are relatively insensitive to this parameter and $\lambda=8$ provides reliable values for these strains. In contrast, radial strain is highly sensitive to the specific value of λ, where lower λ values reduce spatiotemporal smoothness of radial strain but provide more accurate (higher) radial strain values. For radial strain we recommend using λ=3. The temporal weighting factor is μ. If the DENSE images have a very high SNR (i.e., phase SNR > 20), μ could be set to zero. For the general case, our recommendation is $\mu\in\left[ 0.05 , 0.3 \right]$. All figures and results in this manuscript computed by $\lambda=8, \mathrm{and} \mu=0.2$.
