# Supplementary material for: Improved computation of Lagrangian tissue displacement and strain for cine DENSE MRI using a regularized spatiotemporal least squares method
Source: Front Cardiovasc Med. 2023 Mar 16;10:1095159. doi: 10.3389/fcvm.2023.1095159 (PMC10061004; doi:10.3389/fcvm.2023.1095159)
Supplement: Supplementary file 3 [file Presentation_1.pptx]

## Slide 1
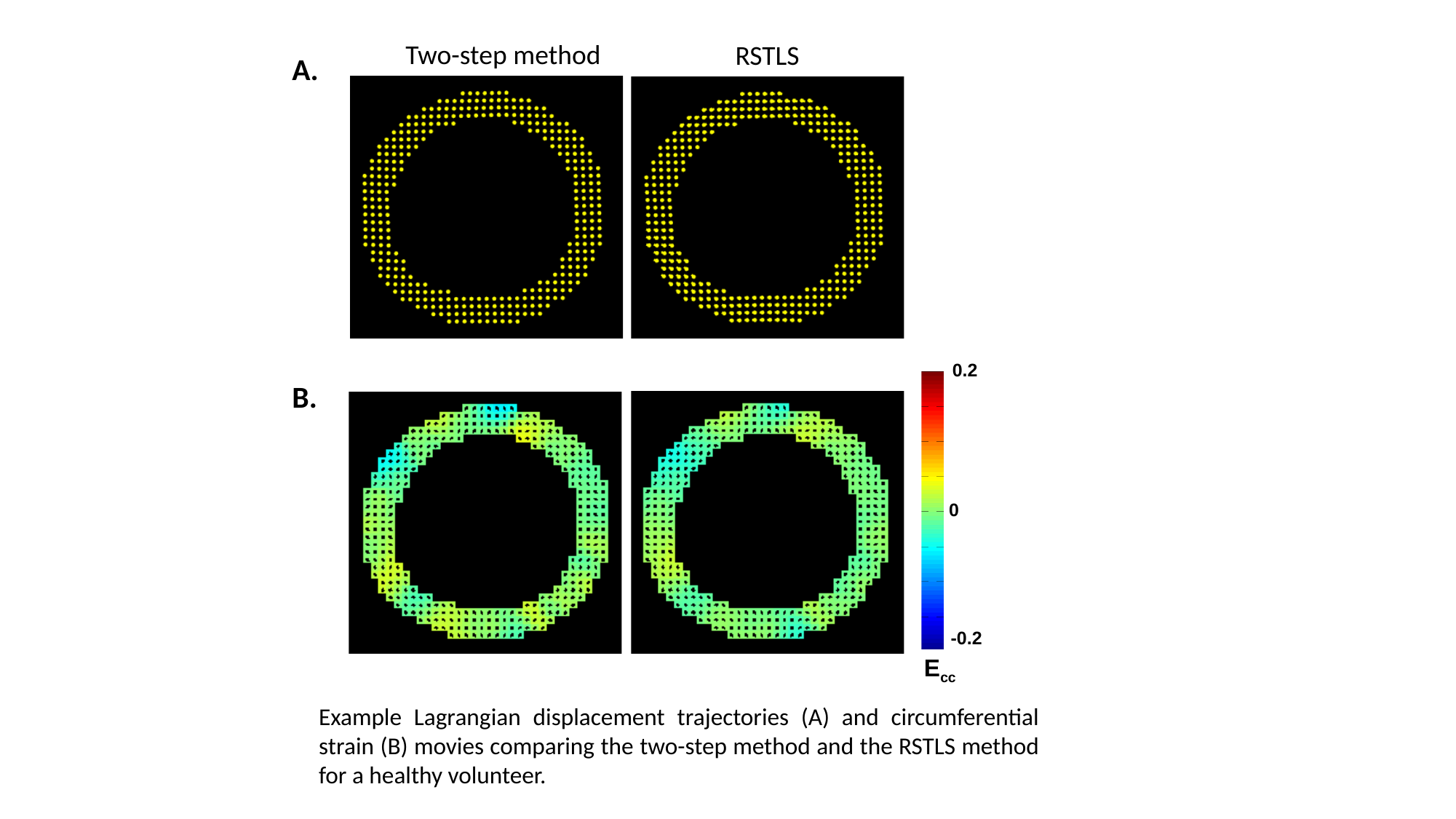

Two-step method
RSTLS
A.
0.2
0
-0.2
Ecc
B.
Example Lagrangian displacement trajectories (A) and circumferential strain (B) movies comparing the two-step method and the RSTLS method for a healthy volunteer.
